# Feasibility of a Brief Intervention to Increase Rapid Primary Care Follow-Up Among African American Patients With Uncontrolled Diabetes

**DOI:** 10.7759/cureus.22756

**Published:** 2022-03-01

**Authors:** Emily M Mylhousen, Elizabeth A Tolley, Satya Surbhi, James E Bailey

**Affiliations:** 1 Center for Health System Improvement, The University of Tennessee Health Science Center, Memphis, USA; 2 Preventive Medicine, The University of Tennessee Health Science Center, Memphis, USA

**Keywords:** diabetes, primary care, medically underserved areas, chronic conditions, uncontrolled diabetes, health care delivery, health care transitions, ambulatory care, implementation science research

## Abstract

The management of diabetes, like many other chronic conditions, depends on effective primary care engagement. Patients with diabetes without a usual source of care have a higher risk of uncontrolled disease, hospitalizations, and early death. Our objective was to study the effect of a brief intervention to help patients in medically underserved areas obtain rapid primary care follow-up appointments following hospitalization. We performed a pilot pragmatic randomized controlled trial of adult patients with uncontrolled diabetes who had been admitted to one of three hospitals in the Memphis, TN, area. The enhanced usual care arm received a list of primary care clinics, whereas the intervention group had an appointment made for them preceding their index discharge. Patients in both groups were evaluated for primary care appointment attendance within seven and fourteen days of index discharge. In addition, we examined barriers patients encounter to receiving rapid primary care follow-up using a secret shopper approach to assess wait times when calling primary care offices. Twelve patients were enrolled with six in each trial arm. Baseline demographics, access to medical care, and health literacy were similar across the groups. Primary care follow-up was also similar across the groups; no improvements in follow-up rates were seen in the group receiving assistance with making appointments. Identified barriers to making primary care follow-up appointments included inability to schedule an urgent appointment, long hold times when calling doctor’s offices and lack of transportation. Additionally, hold times when calling primary care offices were found to be excessively long in the medically underserved areas studied. The study demonstrates the feasibility of providing patient assistance with scheduling rapid primary care follow-up appointments at the time of discharge and the potential to improve care transitions and access to primary care among patients living in medically underserved areas. Larger pragmatic trials are needed to further test alternative approaches for insuring rapid primary care follow-up in vulnerable patients with ambulatory care-sensitive chronic conditions.

## Introduction

Improving health system performance requires improving patient outcomes, reducing avoidable hospitalizations, and decreasing unnecessary spending. Populations with a high disease burden and insufficient access to primary care are at especially high risk of serious medical complications and unnecessary hospitalizations. People with diabetes commonly experience associated comorbidities and complications resulting in high economic and human costs [1, 2]. Access to primary care can reduce emergency department (ED) visits, hospitalizations, and complications for diabetes and other common ambulatory care-sensitive chronic conditions [3, 4]. Previous research demonstrates that engagement in primary care can reduce both prevalence of uncontrolled diabetes and resulting hospitalizations [5]. Thus, diabetes-related hospital admissions are often used to indicate the quality of primary care available to patients [3, 6].

Vulnerable populations living in medically underserved areas present a particular challenge for health system improvement efforts. Patients with multiple complex chronic conditions represent 5% of the population yet account for nearly half of U.S. healthcare expenditures [1]. Evidence suggests that without a usual source of care and adequate primary care engagement, patients with diabetes living in medically underserved areas have a disproportionally higher risk of complications, hospitalizations, and death [7-9].

Given the known importance of primary care in the management of chronic conditions, integrated health systems are testing a variety of approaches to increase primary care engagement and encourage rapid follow-up visits following hospitalization. Previous research has demonstrated some effective approaches for increasing rapid patient follow-up after hospitalizations and visits to the emergency department to decrease avoidable hospitalizations and readmission [10-14]. However, since community health centers are rarely included in hospital-based integrated delivery systems, very limited resources are available to increase rapid follow-up after hospitalization among low-income patients. Lack of studies assessing the effectiveness of the brief, low-cost transition of care approaches for increasing rapid primary care follow-up among vulnerable hospitalized patients represents a critical gap in research. Thus, this pilot study sought to assess: 1) the feasibility of a brief intervention to assist hospitalized patients with uncontrolled diabetes residing in medically underserved areas in obtaining rapid primary care follow-up, 2) patient-reported usual sources of routine/preventive and urgent care and reasons for delaying getting needed care, and 3) the challenges patients face when attempting to schedule follow-up care after hospitalization.

Preliminary results from this study were previously presented as an oral presentation at the 2019 Society of General Internal Medicine Regional Meeting in Houston on February 19, 2019.

## Materials and methods

Design and setting

This study was a three-part study including: 1) a cross-sectional study of patient experience getting needed care, 2) a pilot pragmatic randomized controlled trial and feasibility study of an intervention to improve primary care follow-up, and 3) a secret shopper assessment of the ease of primary care appointment making. This study was conducted in July 2018 within a large nonprofit healthcare delivery system that provides care for medically underserved areas in Memphis, TN. Patients were enrolled during an index hospitalization from one of three adult hospitals and randomized to an: 1) intervention group, or 2) enhanced usual care (control) group. The secret shopper assessment on the difficulty scheduling timely follow-up appointments included clinics in the Memphis area participating in the Patient-Centered Outcomes Research Institute (PCORI)-funded Management of Diabetes in Everyday Life (MODEL) study, a primary care-based pragmatic randomized controlled trial comparing three approaches improving diabetes self-care among African Americans with uncontrolled diabetes [15]. This study was approved by the Institutional Review Board of The University of Tennessee Health Science Center (IRB #18-06080-XP).

Participants and recruitment

Potential participants meeting basic MODEL eligibility criteria were identified using a computerized dashboard to query healthcare delivery system hospital electronic medical records in real-time. Inclusion criteria were: Self-identified African American adults, age > 18 years, uncontrolled diabetes (HbA1C > 8% or random blood sugar > 300mg/dL), have at least one other of 13 chronic health conditions (i.e. hypertension, congestive heart failure, coronary artery disease, cardiac arrhythmias, hyperlipidemia, stroke, arthritis, asthma, cancer, chronic kidney disease, chronic obstructive pulmonary disease, depression, and osteoporosis) using International Classification of Diseases, Tenth Revision, Clinical Modification diagnostic codes, able to provide informed consent, and English speaking. Exclusion criteria were: inability to understand consent procedures, pregnancy, presence of an unstable psychiatric condition or dementia, and perceived unwillingness or inability to participate. In addition, individuals with cognitive impairment were excluded if they experienced difficulty either understanding, following directions, or communicating clearly with program staff. Individuals also were excluded if they exhibited uncontrolled psychiatric symptoms and/or behaviors that may present a danger to program staff or to the study participants themselves.

A research coordinator screened each potential participant to confirm eligibility, explain the study purpose, obtain informed consent, and enroll the participant. After consent, participants completed a baseline survey to assess baseline characteristics and experience getting needed care.

Study interventions

Participants were randomized into one of two groups: an intervention group and an enhanced usual care group. Both groups received a brief list of primary care clinics with addresses and phone numbers as well as a brochure of questions they should ask their primary care provider at a follow-up appointment [16]. Members of the enhanced usual care group were encouraged to schedule their own rapid follow-up appointment with the clinic of their choice within one-to-two weeks of discharge. The intervention group members were asked to identify a clinic and available times within one-to-two weeks of index discharge for a follow-up appointment [16]. The research coordinator then scheduled an appointment on behalf of the participant and provided the participant with a written appointment card with the time, date, address, and phone number of the scheduled appointment. When making patient appointments the research coordinator acted as a “secret shopper” and recorded hold times and inquired about new patient appointment availability.

Measures

Health literacy was evaluated using the validated Chew single item assessment tool employing the question "how confident are filling out medical forms by yourself?" [17,18]. The primary outcome measure was primary care follow-up appointment attendance within 14 days. Patients received a telephone call on 15th day following index discharge to self-report appointment attendance. Secondary outcome measures included: 1) self-reported reasons for delaying needed care (baseline survey), and 2) hold times and new patient appointment availability (secret shopper assessment).

Statistical analysis

Demographic variables and clinical characteristics of the participants randomized to one of two groups were summarized with means and standard deviations for continuous variables and counts and percentages for categorical variables. We used descriptive analyses (proportions and means) to examine baseline characteristics for patients enrolled in the intervention and enhanced usual care groups. For the pilot randomized controlled trial, no bivariate analyses were conducted to examine statistical differences in outcomes between the two groups because of the small sample size. For the secret shopper assessment, bivariate analysis was performed using a chi-square test to compare hold times according to whether the primary care practice employed a call center or not.

## Results

**Figure 1:**
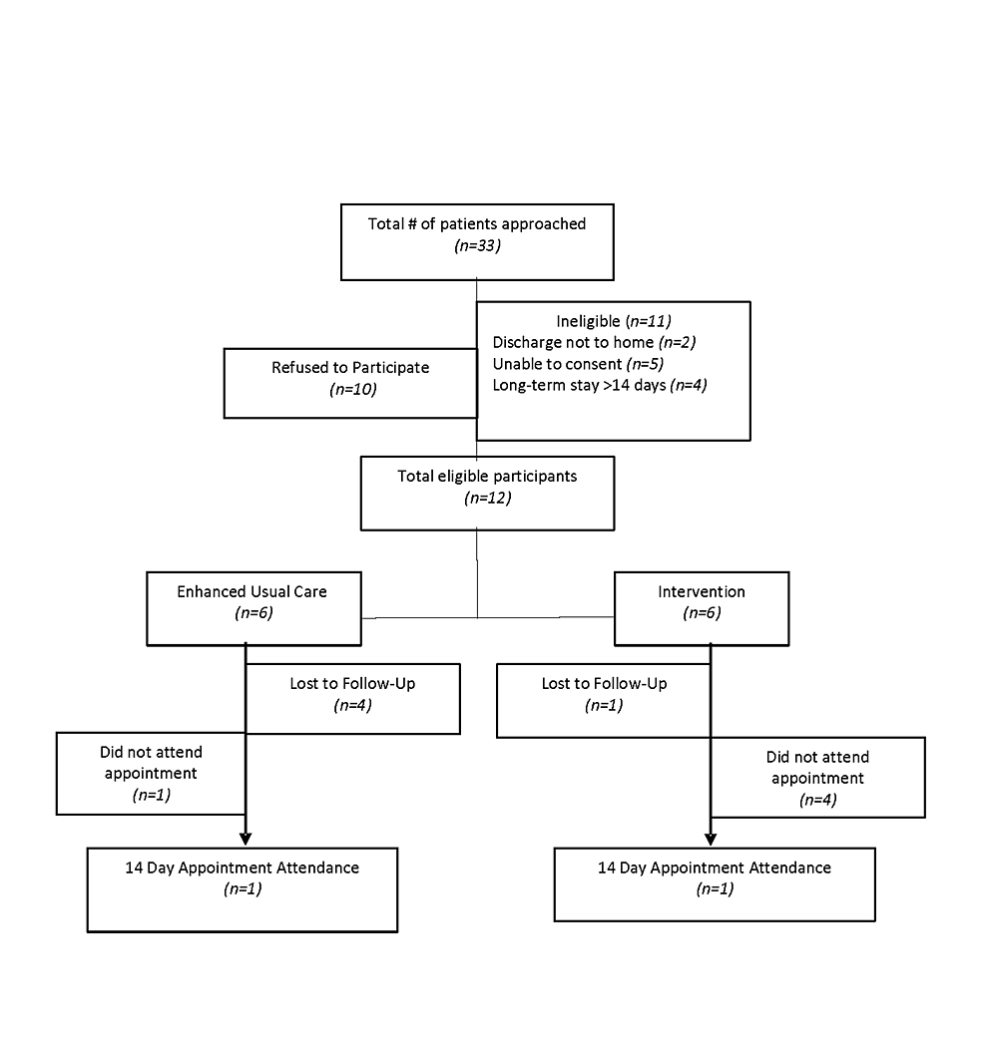
Patient flow diagram

As shown in Figure 1, 33 potentially eligible participants were identified and approached by a researcher during their hospital visit. Of those, 11 (33.3%) were ineligible. Of the remaining 22 patients, 10 (45.5%) refused to participate. The 12 total eligible participants were randomized into the two trial arms, both similar with respect to demographics and clinical characteristics (Table 1). Due to the small sample size, differences between the arms could not be assessed, but more men were enrolled than women overall. The most common chronic diseases other than diabetes was hypertension (75% of participants) and hyperlipidemia (66.7%). Most patients were living with three or more chronic conditions. On average, the participants had been in the hospital two previous times in the past six months. More than half of the patients screened positive for low health literacy (58%) and only 25% of them had a primary care provider. Even so, 66.7% of participants said they regularly go to a clinic, doctor’s office, or health center as their usual source of preventive care.

**Table 1:** Baseline characteristics and reasons participants delayed getting care

Characteristics	Enhanced Usual Care n=6 No. (%)	Intervention n=6 No. (%)
Demographic and Clinical Characteristics of the 12 African American Participants		
Age, year (mean, SD)	54.5 ± 10.7	51.5 ± 10.9
Female	2 (33.3)	2 (33.3)
Single/ Widowed/ Other	2 (33.3)	2 (33.3)
Separated / Divorced	3 (50)	2 (33.3)
High School Education or Less	4 (66.7)	5 (83.3)
Chronic Conditions		
Diabetes	6 (100)	6 (100)
Hypertension	4 (66.7)	5 (83.3)
Hyperlipidemia	4 (66.7)	4 (66.7)
Arthritis	3 (50)	-
Arrhythmias	3 (50)	-
Depression	2 (33.3)	1 (16.7)
# Chronic Conditions (mean, SD)	5 ± 3	3 ±1
Previous Hospitalizations in the past 6 months (mean, SD)	2 ± 2	2 ± 1
Low Health Literacy	3 (50)	4 (66.7)
Has Primary Care Provider	2 (33.3)	1 (16.7)
Usual Source of Preventive Care		
Hospital Emergency Room	1 (16.7)	-
Clinic/Doctor’s Office/ Health Center	3 (50)	5 (83.3)
Does Not Go To One Place Often	2 (33.3)	2 (33.3)
Reasons for Delaying Care		
Couldn’t get through on telephone	1 (16.7)	4 (66.7)
Couldn’t get appointment soon enough	2 (33.3)	4 (66.7)
Has to wait too long to see a doctor	1 (16.7)	4 (66.7)
Clinic/ office wasn’t open	-	1 (16.7)
Didn’t have transportation	2 (33.3)	1 (16.7)

When asked about barriers preventing patients from seeking immediate care when sick, the most common reasons the participants cited were not being able to get through on the telephone and not being able to get an appointment soon enough (Table 1). The proportion of participants in the two treatment arms who followed up with a primary care provider (PCP) within 14 days post-discharge was the same.

Secret shopper assessment involved eight primary care clinics in the Memphis area (Table 2). Of the eight clinics, three clinics had call centers to answer phone calls and make appointments. The other five clinics had receptionists to answer the phone calls and make appointments. The call center clinics had wait times ranging from seven minutes and 12 seconds to 21 minutes and 22 seconds, whereas the clinics without call centers had a maximum hold time of one minute and 45 seconds. Yet all the call center clinics were able to schedule new patient visits within 14-days of call, whereas only 60% of the clinics without call centers were able to make such an appointment (Table 2) (p=0.47).

**Table 2:** Secret shopper assessment of the ease of appointment making

	Clinics with Call Center (n=3)	Clinics without Call Center (n=5)
Hold Times in Minutes and Seconds	7:12 – 21:22	Immediate – 1:45
Accepting New Patients* No. (%) yes	3 (100)	3 (60)

## Discussion

Previous research demonstrates that intensive interventions to increase primary care follow-up have been successful in encouraging timely follow-up after hospitalization [10-14]. However, the current pilot study examines the effectiveness of a simpler, low-cost approach to encourage patients with uncontrolled diabetes to obtain rapid primary-care follow-up after hospitalization discharge. This pragmatic randomized controlled pilot trial found that a simple intervention to assist patients with scheduling a rapid primary care follow-up appointment did not increase follow-up appointment attendance compared with simply providing patients with a list of clinics with phone numbers. The importance of the current results is limited by the study's small size; however, the higher average number of chronic conditions seen in the enhanced usual care arm could have resulted in enhanced usual care patients having higher than expected primary care follow-up appointment attendance. Thus, larger pragmatic trials are needed to determine whether assistance with making rapid primary care follow-up appointments can significantly increase receipt of rapid primary care follow-up and decrease rehospitalization.

Previous research suggests that improving access to care is likely to be more effective in reducing unnecessary hospitalization for ambulatory care-sensitive chronic conditions than is changing patients' propensity to seek health care or eliminating variation in physician practice style [3]. However, the current pilot study clearly highlights the difficulties patients in medically underserved areas face in getting rapid primary care follow-up after hospitalization.

Additionally, we found that long average wait times to schedule appointments and lack of availability of new patient appointments serve as major obstacles to access to essential primary care and chronic disease management. Previous research suggests that without a usual source of care and adequate primary care engagement, patients with diabetes living in medically underserved areas have a disproportionally higher risk of complications, hospitalizations, and death [7-9] However, the current pilot suggests that barriers to such primary care after hospitalization are substantial. Specifically, access to timely primary care follow-up following hospitalization is critical to mitigating the burden of diabetes and other ambulatory care-sensitive chronic conditions. Larger pragmatic clinical trials can help elucidate these barriers and demonstrate effective ways to address them so that people with ambulatory care sensitive chronic conditions in medically underserved areas get the essential primary and preventive care they need most.

## Conclusions

This pilot study confirmed that hospitalized patients with uncontrolled diabetes have great difficulty getting needed outpatient follow-up care. This pilot study demonstrated very low rates of rapid primary care follow-up after hospitalization among patients with uncontrolled diabetes even among those receiving intensive assistance with appointment scheduling. Vulnerable patients in medically underserved areas commonly experience difficulty getting through on the phone, due to excessive hold times, as well as long wait times for follow-up appointments and other difficulties including finding transportation. Furthermore, this pilot study documented that hold times for scheduling primary care visits in medically underserved areas are long and serve as a major barrier to rapid primary care follow-up post-hospitalization. The substantial barriers to getting rapid primary care follow-up faced by patients in medically underserved areas face may be insurmountable without systems-level integrated care solutions. Our data indicate that hospital assistance with making appointments is not likely a panacea, since, despite assistance, appointment attendance showed no improvement. Additional investigation is needed to explore ways to alleviate the difficulty patients face scheduling follow-up primary care after hospital discharge and to determine whether pre-discharge scheduling of follow-up appointments can improve patients’ appointment attendance. This study suggests that primary care capacity, accessibility, and integration with hospital networks are fundamental problems that must be addressed to adequately serve our most vulnerable patients.
